# CEP55 Promotes Cell Motility via JAK2–STAT3–MMPs Cascade in Hepatocellular Carcinoma

**DOI:** 10.3390/cells7080099

**Published:** 2018-08-08

**Authors:** Minjing Li, Ju Gao, Defang Li, Yancun Yin

**Affiliations:** 1 Taishan Scholar Immunology Program, School of Basic Medical Sciences, Binzhou Medical University, Yantai 264003, China; liminjing512@126.com; 2 Medicine and Pharmacy Research Center, Binzhou Medical University, Yantai 264003, China; 3 Departments of Pathology, Case Western Reserve University, Cleveland, OH 44106, USA; jxg712@case.edu; 4 Department of Clinical College of Chinese and Western Medicine, Binzhou Medical University, Yantai 264003, China; lidefang@163.com

**Keywords:** CEP55, hepatocellular carcinoma, JAK2-STAT3, migration, invasion

## Abstract

Hepatocellular carcinoma (HCC) is one of the most common malignancies and has a poor prognosis. Novel diagnostic or prognostic biomarkers and potential therapeutic targets for HCC are thus urgently needed. CEP55 plays a crucial role in regulating physical cytokinesis. Whether, and how, CEP55 contributes to HCC development remains unclear. Herein, we demonstrate that CEP55 is abnormally upregulated in HCC tissue, and these high levels of CEP55 are closely related to the poor prognosis of HCC patients. Knockdown of CEP55 expression significantly inhibits HCC cell migration and invasion. We also demonstrate that CEP55 physiologically interacts with JAK2 and promotes its phosphorylation; thus, it is a novel regulator of JAK2–STAT3 signaling and its target genes MMP2/9. Finally, blocking JAK2 or STAT3 blunts the stimulation of migration and invasion due to CEP55 overexpression. In summary, our results suggest that CEP55, as an oncogene, promotes HCC cell migration and invasion through regulating JAK2–STAT3–MMPs signaling.

## 1. Introduction

Hepatocellular carcinoma (HCC) is a high mortality type of primary liver cancer. It is the most common malignancy worldwide, especially in Asia, Africa, and Southern Europe. It is the fifth most common aggressive cancer and the second leading cause of cancer death [1]. During 2012, there were approximately 782,500 new liver cancer cases and 745,500 deaths worldwide, and about 50% of the total cases and deaths were from China. In addition, the incidence of HCC in areas with historically low rates of HCC, such as Western Europe and North America, have increased [2]. This emphasizes the urgency of establishing new therapeutic targets for successful intervention.

Intrahepatic and extrahepatic metastasis is a pivotal step in determining invasive phenotypes and is a major cause of poor prognosis and high relapse in HCC. Cell migration and invasion are the key parameters for the metastatic spread of cancer cells. A complex array of molecules and signaling pathways influences the migration and invasion potential of HCC cells. To be metastatic, cancer cells must upregulate the expression of a variety of metastasis-promoting genes. However, the underlying molecular mechanism that mediates cell migration and invasion remains largely unknown. In-depth understanding of the mechanisms may facilitate the development of effective metastasis-targeted therapy and the improvement of the overall prognosis of HCC patients.

CEP55, the most recently discovered member of the centrosome relative protein family, plays key roles in cell division [3,4]. Throughout mitosis, CEP55 remains associated with the centrosome and is recruited to the mitotic spindle [4]. At the terminal stage of cytokinesis, CEP55 is also required for mid-body structure and the completion of cytokinesis [5]. The overexpression of CEP55 results in cytokinesis defects and an increase in multinucleated cells, which may cause tumorigenesis [6]. Recent studies have found that elevated expression of CEP55 is significantly associated with tumor stage, invasiveness, metastasis, and poor prognosis in multiple tumor types [6,7,8,9,10,11,12,13]. However, it is still unclear whether and how CEP55 regulates the invasion and metastasis of HCC.

In this study, our data indicate that CEP55 is overexpressed in HCC and that high levels of CEP55 are associated with a poor prognosis in HCC patients. Moreover, CEP55 promotes the migration and invasion of HCC cells. Knockdown of CEP55 expression significantly decreases the cell motility of HCC cell motility via suppressing the JAK2–STAT3–MMPs signaling axis. These results indicate that targeting CEP55 may be effective in the treatment of HCC.

## 2. Materials and Methods

### 2.1. Reagents and Antibodies

The following antibodies were used: anti-CEP55 (#23891-1-AP), MMP2 (#66366-1-Ig), MMP9 (#10375-2-AP), STAT3 (#60199-1-Ig), JAK1 (#66466-1-Ig), JAK2 (#17670-1-AP), and Actin (#60008-1-Ig) (Proteintech Group, Chicago, IL, USA); and p-JAK1 Tyr1034/1035 (#74129), p-JAK2 Tyr1007/1008 (#3771), and p-STAT3 Tyr705 (#9145) (Cell Signaling Technology, Beverly, MA, USA). The biological reagent IL-6 was acquired from Sigma Chemical (St. Louis, MO, USA). JAK2 inhibitor XL019 (#S7036), STAT3 inhibitor C188-9 (#S8605) was from Selleck (Shanghai, China).

### 2.2. Gene Expression and Survival Analysis

The expression levels of CEP55 mRNA in human HCC tumors and normal tissues were determined with GENT (Gene Expression across Normal and Tumor Tissue) databases, as previously mentioned [14]. We analyzed the publicly available gene expression datasets from human cancer research for a survival analysis, as described previously [15]. The data were obtained from the Cbioportal TCGA provisional set. (http://www.cbioportal.org/) (Supplementary File 2). A Kaplan–Meier survival analysis was conducted in which the HCC patients were divided into two groups at the 50th percentile based on log2-transformed CEP55 mRNA expression.

### 2.3. Quantitative Real-Time PCR

Total RNA was extracted with Trizol Reagent (Invitrogen, Carlsbad, CA, USA). The cDNAs were reverse transcribed using the AMV reverse-transcription kit (Promega Biotechnology, Madison, WI, USA). CEP55 and GAPDH were amplified by real-time PCR using a SYBR Green Real-Time PCR reagent (Life Technologies, Carlsbad, CA, USA). The primer sequences for human CEP55 were F: 5′-TGAGAGACCAACTGAAGGCCA-3′; R: 5′-GCTTTCAACACCTGCTCCCTC-3′. The primer sequences for human GAPDH were F: 5′-AACTTTGGCATTGTGGAAGGA-3′; and R: 5′-AACATCATCCCTGCTTCCAC-3′ (reverse). Quantitative PCR reactions were performed under the following conditions: 5 min at 96 °C, followed by 35 cycles at 95 °C for 15 s and at 60 °C for 60 s. A comparative CT method (2^−∆∆CT^) was applied to quantify gene expression.

### 2.4. Lentivirus Construction and Infection

The lentiviral vector ZsRed was used to construct exogenous CEP55. The GFP tagged lentiviral vector PLL3.7 was used to knockdown the expression of CEP55, JAK2, and Stat through shRNA designed to target each gene. The shRNA targeted sequences were as follows:

- CEP55 shRNA1: 5′-ACCTCACGAATTGCTGAAC-3′; CEP55 shRNA2: 5′-GACTGAGAGACCAACTGAA-3′;
- CEP55 shRNA3: 5′-GCTCCAAACTGCTTCAACT-3′; CEP55 shRNA4: 5′-CTGAAAGATGCTCTGGAGA-3′; and
- JAK2 shRNA: 5′-GCAGAATTAGCAAACCTTATA-3′; STAT3 shRNA: 5′-TGTTCTCTATCAGCACAAT-3′.

To determine the lentiviral supernatant production, PLL3.7-based shRNA constructs or the ZsRed-based CEP55 overexpression construct were mixed with envelope plasmid psPAX2 and pMD2G (7.5 μg:5.5 μg:2 μg). Then, the mixture was transfected into HEK-293T cells using polyJet regent. The lentivirus-containing supernatant was harvested at 48 h and 72 h post-transfection, respectively. The lentivirus-containing supernatant was used to infect HCC cells after filtration with a 0.45-μm filter. For infection, HCC cells were seeded into 6-well plate and grown to ~50% confluence. Then, the cells were maintained in 5 mL lentivirus supernatant for 6 h, before being discarded and replaced with DMEM medium with 10% FBS. Twenty-four hours later, the cells were infected for the second time.

### 2.5. Western Blot

The cultured cells were lysed with pre-cooling RIPA lysis buffer with PMSF and aprotinin. Then, the mixture was incubated on ice for 30 min and centrifuged for 15 min at 14,000× *g*, and the supernatant was transferred to a new EP tube. The supernatant was harvested and analyzed for protein concentration using a BCA kit (Thermo Fisher Scientific, Waltham, MA, USA). Aliquots of protein were boiled in 1× loading buffer for 10 min. Samples containing 30 μg of total protein were resolved by 10% SDS-PAGE and proteins were transferred to a PVDF membrane (Millipore Corporation, Billerica, MA, USA). Each membrane was blocked with 5% non-fat dry milk in 1× TBST for 1 h at room temperature. Then, the membrane was incubated with a primary antibody overnight at 4 °C and a secondary antibody for 1 h at room temperature. The membrane was detected by chemiluminescent agents after being washed three times with 1× TBST for 30 min. To ensure that equal amounts of the sample protein were applied for electrophoresis, actin was used as an internal control. The protein level was analyzed by densitometric analysis

### 2.6. Co-Immunoprecipitation (Co-IP) Assay

Cells were infected with flag-CEP55 for 48 h, and then cells were lysed with cold lysis buffer (1% Triton X-100, 40 mM Hepes pH 7.5, 120 mM NaCl, 1 mM EDTA, 10 mM pyrophosphate, 10 mM glycerophosphate, 50 mM NaF, 0.5 mM orthovanadate, PMSF, and aprotinin (Roche, Indianapolis, IN, USA)). The supernatant (1 mg total protein) was used for immunoprecipitation with the indicated mouse monoclonal antibody (Flag, # 66008-2-Ig, (Proteintech, Chicago, IL, USA); CEP55, # SAB1409483, Sigma, St. Louis, MO, USA; or JAK2, # AHO1352, Thermo Fisher, Waltham, MA, USA) or mouse IgG at 4 °C overnight. Then, the supernatant was incubated with 40 μL of protein G-agarose beads with rotation for 3 h at 4 °C. After three washes, immunoprecipitated proteins were immunoblotted.

### 2.7. Cell Wound Scratch Assay

HCC cells were infected with shScr (scramble shRNA) or shRNA targeting CEP55 in six-well plates. At 24 h post-infection, the plate bottom of the cell culture was scratched with a 1-mm opening using a pipette tip and washed two times with PBS. The opening width was imaged under a phase-contrast microscope every 6 h. In order to ensure the consistency of the observations, different observation points were marked along the gap, and the same field was observed each time. The opening width was measured, and the relative migrated distance was calculated.

### 2.8. Trans-Well Assay

HCC cells were infected with shScr or shRNA targeting CEP55. Twenty-four hours after infection, the cells were harvested and counted, and then 3 × 10^4^ cells were transferred to the upper chamber in DMEM medium without serum. Later on, 750 μL of chemoattractant was added to the basal chamber and incubated for eight hours. Then, the cells beneath the upper chambers were fixed, stained, and counted.

### 2.9. Statistical Analysis

All experiments were repeated three times with similar results. A representative experiment is shown. The analysis was conducted with SPSS 17.0 software. All results are presented as means ± SDs. Student’s *t*-tests were performed to compare the difference between two groups. One-way ANOVA tests were performed to compare differences among three or more groups. The log-rank test was performed to compare the survival difference between the CEP55 high and low groups. Fisher’s exact tests were performed to analyze the relationships between CEP55 mRNA expression and clinicopathologic features. The differences were considered statistically significant if *p* < 0.05.

## 3. Results

### 3.1. CEP55 Expression Is Upregulated in HCC Tissues and Cell Lines

**Figure 1:**
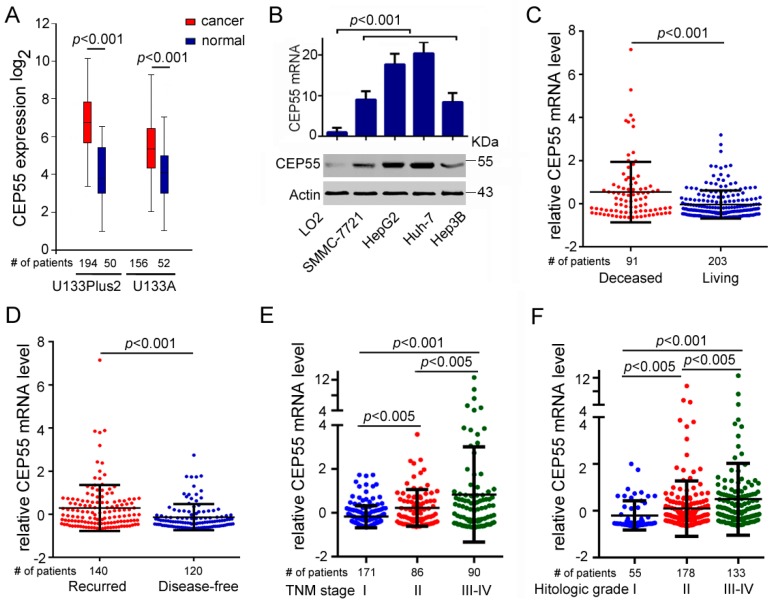
Elevated expression of CEP55 in hepatocellular carcinomas (HCCs). (**A**) Fold changes of the CEP55 mRNA expression level in normal or HCC liver tissues. Data were obtained from the GENT (gene expression across normal and tumor tissue) database; (**B**) Upper panel: RT-PCR analysis was used to determine the relative mRNA expression of CEP55 in the indicated cell lines. Lower panel: western blot analysis was used to determine the relative protein expression of CEP55 in the indicated cell lines; (**C**) Log2-transformed CEP55 mRNA expression levels in the deceased or living HCC samples; (**D**) Log2-transformed CEP55 mRNA expression levels in the recurring or disease-free HCC samples; (**E**) Log2-transformed CEP55 mRNA levels in HCC patients with different tumor-node-metastasis (TNM) stages; (**F**) Log2-transformed CEP55 mRNA levels in HCC patients with different histologic grades. ((**C**–**F**): Data were obtained from Cbioportal, liver hepatocellular carcinoma (TCGA, provisional)).

To confirm the effects of CEP55 on HCC, we performed an in silico analysis to determine the expression level of CEP55 in HCC samples and normal livers using data from GENT. The results showed that the expression of CEP55 in HCC samples was significantly higher than that in corresponding normal tissues (Figure 1A). Furthermore, we detected higher CEP55 expression in HCC cell lines such as Hep3B, Huh-7, HepG2, and SMMC-7721 in comparison to immortalized hepatocytes LO2 cells (Figure 1B). In particular, Huh-7 and HepG2 cells exhibited the highest expression levels of CEP55 compared with other HCC cells evaluated both in transcription and protein level (Figure 1B). In addition, the expression of CEP55 was shown to be significantly elevated in HCC tumor tissues of deceased patients compared with the HCC tissues of living patients (Figure 1C). Additionally, recurring HCC patients also showed higher expression of CEP55 than disease-free patients (Figure 1D). Importantly, the expression of CEP55 increased gradually along with progression of HCC from tumor-node-metastasis (TNM) stages I to IV (Figure 1E). Furthermore, CEP55 expression increased as the histologic grade of HCC patients increased (Figure 1F). These data demonstrate that CEP55 is highly expressed in HCC cells and may support HCC propagation.

### 3.2. Overexpression of CEP55 Is a Poor Prognostic Factor for HCC Patients

**Figure 2:**
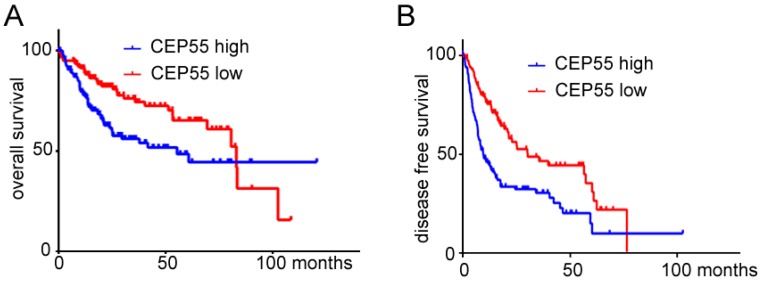
The prognostic effects of high and low expression of CEP55 in HCC patients. (**A**,**B**) Expression data of CEP55 and clinical data of HCC patients were obtained from Cbioportal. Patients were separated into two groups equally based on log2-transformed expression of CEP55, and % overall survival (**A**); or disease-free survival (**B**) vs. time was plotted. For the OS curves, N = 294, Log-rank test *p* = 0.0048, HR = 1.817 (1.200–2.735); for the DFS curves N = 260, Log-rank test *p* < 0.0001, HR = 2.090 (1.520–2.972).

To confirm whether the elevated expression of CEP55 in HCC tissues and cell lines correlated with clinical indicators, we analyzed the correlation between the expression levels of CEP55 mRNA and the clinicopathological features of HCC patients (Table 1). CEP55 expression was obviously related to the level of serum AFP (*p* < 0.0001), vascular invasion (*p* = 0.0095), histologic grade (*p* < 0.0001), and TNM stage (*p* = 0.0200) in HCC patients. To clarify the relationship between CEP55 expression and clinical outcome in HCC patients, a Kaplan–Meier analysis of the association between CEP55 expression and the clinical endpoint of HCC patients was performed. The results showed that high expression of CEP55 in HCC patients was markedly related to shortened overall survival (OS, *p* = 0.0048, HR = 1.817) (Figure 2A) and disease-free survival (DFS, *p* < 0.0001, HR = 2.090) (Figure 2B). These data indicate that CEP55 can support the progression of HCC and may be an effective biological marker of poor outcomes in HCC patients.

### 3.3. CEP55 Knockdown Suppresses the Motility Activity of HCC Cells

**Figure 3:**
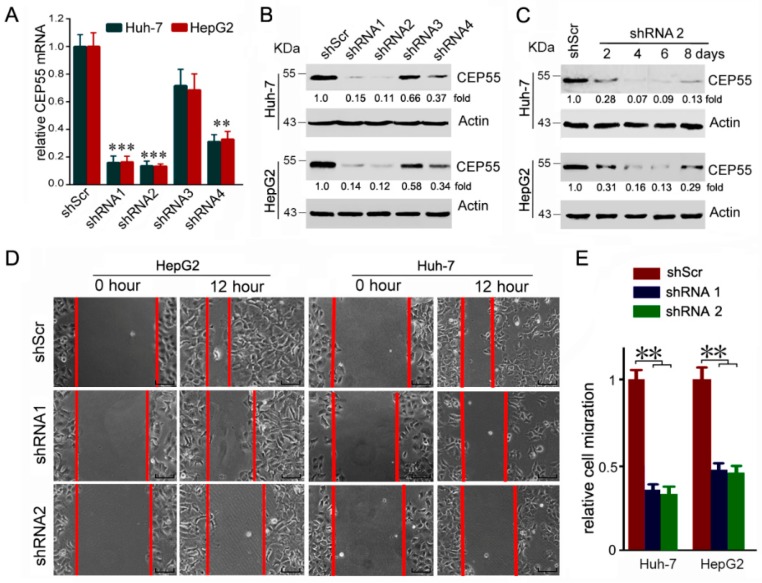

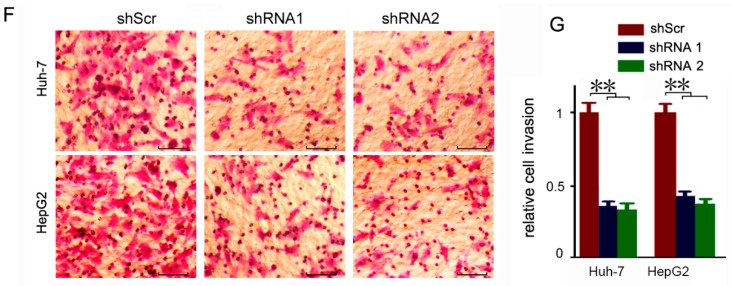
The motility of HCC cells is inhibited by CEP55 shRNAs. (**A**) HCC cells were infected with the designed shRNAs. Then transcription of CEP55 was analyzed by real-time PCR at 24 h post-infection. N = 4, ** *p* < 0.005; *** *p* < 0.001 compared to the scramble group; (**B**) HCC cells were infected with designed shRNAs. Then, CEP55 expression was analyzed by Western blotting at 72 h post-infection. A representative of three experiments is shown. The densitometric quantification is presented as the fold change compared to actin; (**C**) HCC cells were infected with shRNA2, followed by an analysis of CEP55 expression by Western blotting at the different indicated times post-infection. A representative of three experiments is shown. The densitometric quantification is presented as the fold change compared to actin; (**D**) Cells were infected with the indicated shRNA lentivirus. The gap width at multiple constant points was measured at 12 h post-infection. Representative images are plotted. Bars, 50 μm; (**E**) The relative migration distance of HCC cells compared to the control group is plotted. N = 4, ** *p* < 0.005; (**F**) Representative images of trans-well analyses to evaluate the invasion ability of HCC cells transfected with indicated shRNAs. Bars, 50 μm; (**G**) A summary of relative invasion cell numbers compared to the control group is plotted. N = 4, ** *p* < 0.005.

To further explore the biological function of CEP55, a loss-of-function approach mediated by shRNAs was used to inhibit CEP55 expression in Huh-7 and HepG2 cells which exhibited a high expression level of CEP55 in the evaluated cells. The results showed that the three pairs of shRNAs 1, 2 and 4 were able to suppress CEP55 expression more efficiently than shScr at both the mRNA and protein levels (Figure 3A,B). Specifically, the expression level of CEP55 was reduced to <15% of shScr by shRNA1 and shRNA2 (Figure 3A). In addition, we assessed the time-dependent knockdown efficiency of shRNA2 at the protein level. The results showed that shRNA2 was sufficient to keep CEP55 at a relatively low level for more than one week (Figure 3C). Thus, we chose shRNA1 and shRNA2 for the next function studies due to their higher knockdown efficiency. Since high expression of CEP55 is significantly associated with the TNM stage and vascular invasion in HCC patients, we next detected the potential role of CEP55 in HCC cell migration and invasion. In the wound healing assays, cells in the CEP55-deficient group migrated more slowly than in the shScr-treated group (Figure 3D,E). Then, we used the trans-well assays to determine the effect of CEP55 on HCC cell invasion. We found that downregulation of CEP55 by shRNA1 or shRNA2 resulted in a sharp decline in the invasiveness of Huh-7 and HepG2 cells (Figure 3F,G).

Since CEP55 plays significant roles in cell mitosis and proliferation, the wound healing and trans-well assays were completed within 12 h in order to avoid the disturbance of cell proliferation. In fact, our data indicated that knockdown expression of CEP55 did not reduce cell proliferation compared to control cells within 12 h (Figure S1). This result further supports the conclusion that CEP55 promotes HCC cell motility independently of increased cell proliferation. Together, these results suggest that CEP55 supports HCC cell migration and invasion in vitro.

### 3.4. CEP55 Promotes Expression of MMPs in HCC Cells

**Figure 4:**
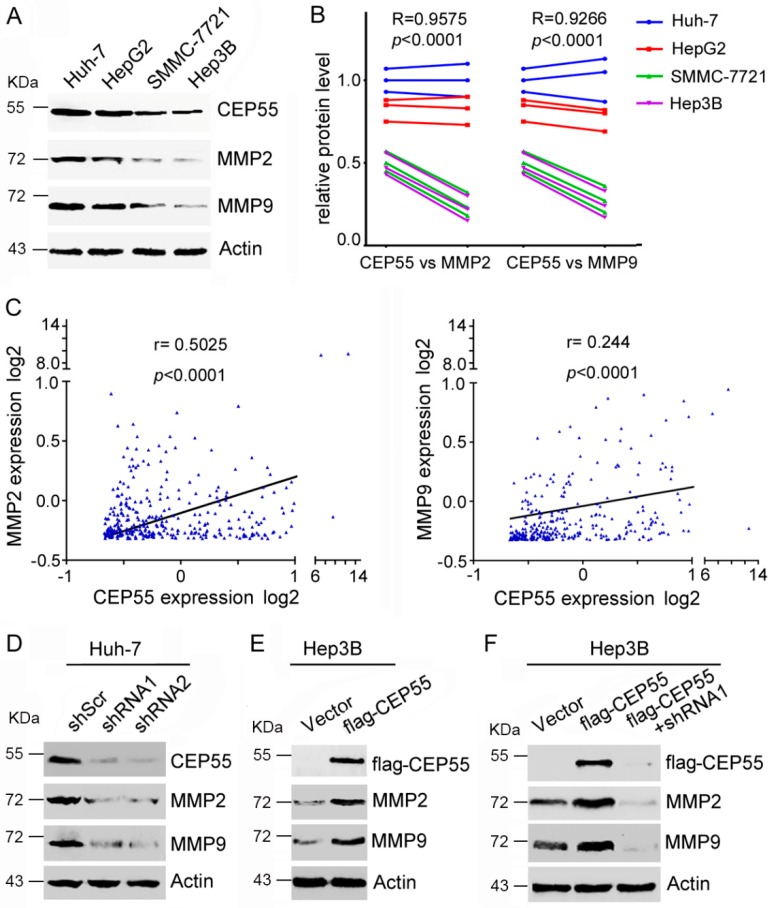
CEP55 promotes the expression of MMP2 and MMP9 in HCC cells. (**A**) The expression of CEP55, MMP2, and MMP9 in the indicated cell lines was analyzed by western blotting. A representative of three independent experiments is shown; (**B**) A densitometric analysis of CEP55 and MMPs was performed with Image J. The expression of CEP55, MMP2, and MMP9 in Huh7, HepG2, smmc-7721, and Hep3B cells showed highly significant positive correlations; (**C**) The expression of CEP55, MMP2, and MMP9 in 373 HCC patients showed highly significant positive correlations (data from Cbioportal); (**D**) Cells were infected with indicated shRNA for 72 h, followed by a western blot analysis of the expression of MMPs; (**E**) Flag-tagged CEP55 was overexpressed in Hep3B cells for 72 h. The expression of MMPs was analyzed; (**F**) Flag-tagged CEP55 was pre-infected into Hep3B cells for 24 h, followed by treatment with shRNA 1 for 72 h. The expression of MMP was analyzed.

Previous studies have clearly shown that the epithelial–mesenchymal transition (EMT) is closely related to the invasion and migration of cancer cells [16]. In particular, several studies have shown that CEP55 plays an important role in regulating EMT in oral cavity squamous cell carcinoma [8], lung cancer [9], and ovarian cancer [12]. To explain the correlation between CEP55 and EMT processes in HCC cells, we detected the changes in the EMT markers between CEP55-shRNAs and the control group. The analyses revealed that the inhibition of CEP55 has no effect on the transcription of N-cadherin and E-cadherin (*p* > 0.05), although there is a slight downregulation of N-cadherin and upregulation of E-cadherin in CEP55 shRNA treated cells (Figure S2). To further elucidate the possible CEP55-mediated signaling pathways involved in the control of HCC cell motility, the molecular target—matrix metalloproteinases (MMPs), which are significantly associated with migration and invasion regulation—were evaluated by western blot analysis. Higher expression of MMP2 and MMP9 was observed in Huh-7 and HepG2 cells than that in SMMC-7721 and Hep3B cells, suggesting that expression of MMPs is positively correlated with the CEP55 level (Figure 4A,B). Moreover, Spearman’s rank tests showed that the mRNA expression of CEP55 was significantly and positively correlated with the mRNA expression of MMP2 (R = 0.5025, *p* < 0.0001) and MMP9 (R = 0.244, *p* < 0.0001) in paired human HCC samples (Figure 4C). Furthermore, MMP expression was tested in Huh-7 cells that were infected with CEP55 shRNAs. Results demonstrated that knockdown CEP55 expression inhibited the expression of MMP2 and MMP9 (Figure 4D). Conversely, overexpression of CEP55 promoted MMP2 and MMP9 expression in Hep3B cells (Figure 4E). Interestingly, the upregulated expression of MMPs induced by CEP55 overexpression could be inhibited by CEP55 shRNA (Figure 4F). These observations suggest that CEP55 may regulate MMP expression.

### 3.5. CEP55 Stimulates the JAK2–STAT3–MMP Axis in HCC Cells

**Figure 5:**
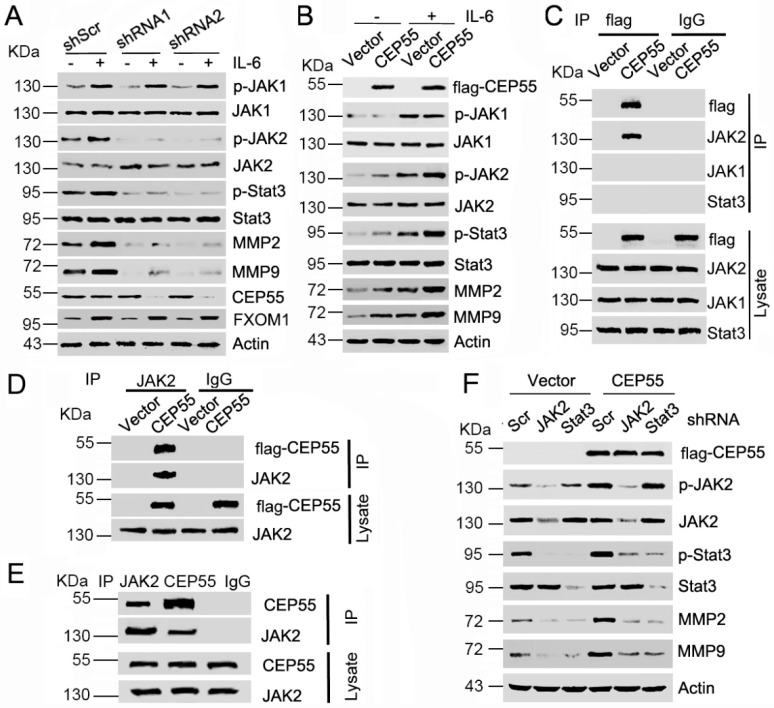
CEP55 promotes MMP expression via activating JAK2–STAT3 signaling. (**A**) Huh-7 cells were pre-infected with the indicated CEP55 shRNA for 72 h, and then cells were incubated with or without 20 ng/mL IL-6 for 30 min, followed by an analysis of the expression of the indicated proteins by western blotting; (**B**) Flag-tagged CEP55 was pre-transfected into Hep3B cells for 72 h, and then cells were incubated with or without 20 ng/mL IL-6 for 30 min, followed by an analysis of the expression of the indicated proteins by western blotting; (**C**) Vector or Flag-CEP55 plasmids were transfected into HepG2 cells. JAK2 was immunoprecipitated with anti-flag antibody; (**D**) Vector or flag-CEP55 plasmids were transfected into Hep3B cells. Ectopic CEP55 was immunoprecipitated with anti-JAK2 antibody; (**E**) Immunoprecipitation of CEP55, JAK2, and IgG in HepG2 cells, and western blotting analysis of the indicated proteins; (**F**) Flag-tagged CEP55 was pre-transfected into Hep3B cells for 24 h, followed by treatment with the control, JAK2, and STAT3 shRNA for 48 h. Expression levels of the indicated proteins were analyzed by western blotting.

Multiple extracellular factors—such as cytokines, growth factors, and interactions with adjacent cells—could regulate the expression of MMPs during cancer cell migration and invasion. Earlier studies reported that IL-6 promotes MMP2 [17] and MMP9 [18] expression by facilitating the JAK/STAT3 signaling pathway. Herein, we speculate that CEP55 may regulate IL-6 and its downstream signaling pathways and may be related to the phosphorylation and activation of STAT3. To determine the mechanism underlying CEP55-regulated expression of MMPs, we first infected Huh-7 cells with CEP55 or Scramble shRNAs and then treated them with IL-6. The western blotting results showed that IL-6 induced expression of MMP2, MMP9, and FXOM1 in control cells; however, CEP55 knockdown attenuated the IL-6-induced expression of MMPs but not FXOM1 (Figure 5A). Interestingly, CEP55 knockdown weakened the IL-6-induced phosphorylation of JAK2 and STAT3, but not JAK1 (Figure 5A). Conversely, overexpression of CEP55 promoted IL-6-induced phosphorylation of JAK2 and STAT3, and increased IL-6 induced expression of MMP2 and MMP9 (Figure 5B). The abovementioned results imply there is a functional interaction between CEP55 and JAK2. Interestingly, the co-IP results showed that the flag antibody co-immunoprecipitated JAK2, but not JAK1 or STAT3, suggesting that CEP55 interacts with JAK2 (Figure 5C). Reciprocally, the JAK2 antibody co-immunoprecipitated ectopic CEP55 in Hep3B cells (Figure 5D). Importantly, the CEP55 antibody co-immunoprecipitated JAK2 in HepG2 cells. Additionally, the JAK2 antibody co-immunoprecipitated endogenic CEP55 (Figure 5E). These data indicate that CEP55 physically interacted with JAK2. Furthermore, knockdown expression of JAK2 or STAT3 attenuated CEP55 overexpression-induced expression of MMP2 and MMP9 (Figure 5F). These results seem to imply that CEP55 regulates MMP expression via stimulation of the JAK2/STAT3 pathway in HCC cells.

### 3.6. JAK2 or STAT3 Blocking Abrogates the Pro-Migration and Pro-Invasion Effects of CEP55

**Figure 6:**
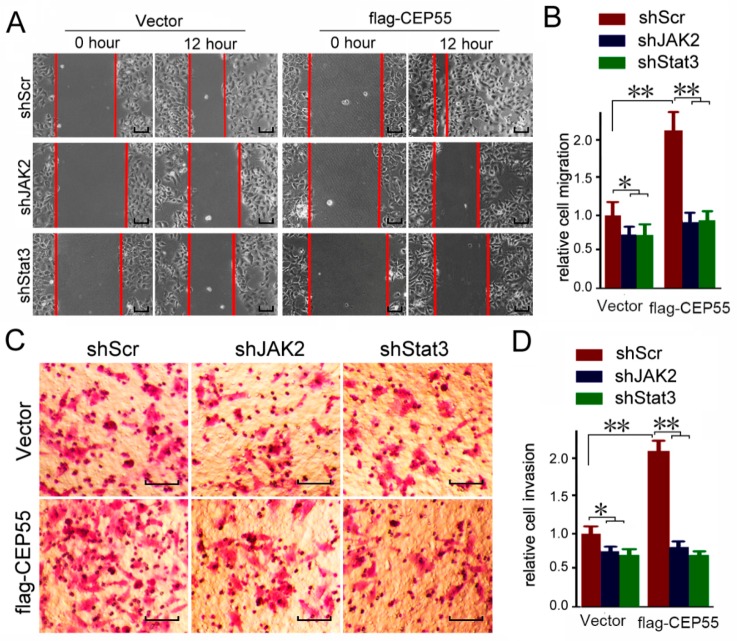

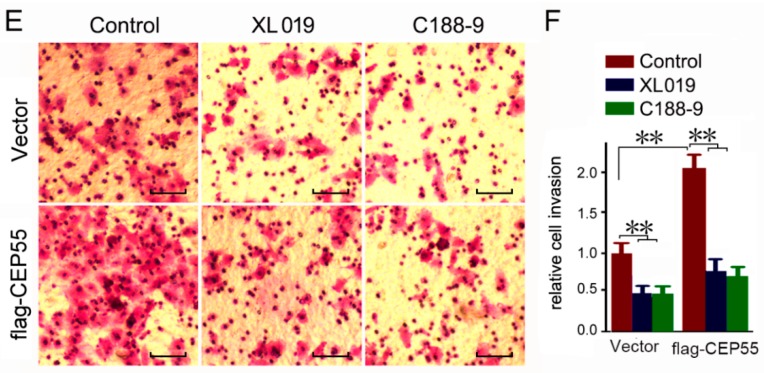
Knockdown of JAK2 or STAT3 expression abrogates the pro-migration and -invasion effects on CEP55 overexpression. (**A**) Hep3B cells were pre-infected with flag-CEP55 for 24 h, and then were infected with indicated shRNA for 48 h. Cells’ migration ability was determined by wound healing assays. Representative microscope images are plotted. Bars, 50 μm; (**B**) A plot of the summary of the relative migration distance. The control group was set as 1. N = 4, * *p* < 0.005; ** *p* < 0.001; (**C**) Hep3B cells were pre-infected with flag-CEP55 for 24 h and were then infected with the indicated shRNA for 48 h. Cells’ invasion ability was determined by trans-well assays. Representative microscope images are plotted. Bars, 50 μm; (**D**) A plot of the summary of the relative invasion cell number. The control group was set as 1. N = 4, * *p* < 0.005; ** *p* < 0.001; (**E**) Hep3B cells were pre-infected with flag-CEP55 for 24 h and were then infected with 10 nM JAK2 inhibitor XL019 or 10 nM STAT3 inhibitor C188-9 for 48 h. Cells’ invasion ability was determined by trans-well assays. Representative microscope images are plotted. Bars, 50 μm; (**F**) A plot of the summary of the relative invasion cell number. The control group was set as 1. N = 4, ** *p* < 0.001.

The abovementioned data indicated that CEP55 promotes the activation of JAK2–STAT3 signaling. To determine whether JAK2 and STAT3 are involved in the CEP55 promoted migration and invasion of HCC cells, vector or flag-CEP55 plasmids were pre-transfected into Hep3B cells and then treated with JAK2 or STAT3 shRNA. Cell migration was detected through a wound-healing assay. In the wild-type Hep3B cells, JAK2 or STAT3 knockdown slightly suppressed cell migration. Overexpression of CEP55 significantly enhanced the migration of Hep3B cells. Interestingly, blocking JAK2 or STAT3 blunted the stimulation of migration by CEP55 overexpression (Figure 6A,B). In addition, blocking JAK2 or STAT3 blunted the stimulation of invasion by CEP55 (Figure 6C,D). Consistent with the results that CEP55 has no effects on JAK1 activation, blocking JAK1 did not affect CEP55 overexpression-induced cell invasion (Figure S3). Furthermore, blocking JAK2 or STAT3 by chemical inhibitor also blunted the stimulation of invasion by CEP55 overexpression (Figure 6E,F). Together, the abovementioned data indicate that the oncogenic biological function of CEP55 may be mediated by the JAK2/STAT3 signaling pathway. In summary, these results indicate that CEP55 supports HCC progression through the promotion of JAK2/STAT3 signaling activation and induction of MMP expression.

## 4. Discussion

Tumor metastasis, invasion, and recurrence are the main causes of death in human HCC patients. The development of anti-HCC drugs is a daunting challenge because HCC is highly invasive, and the liver can show severe adverse effects. Thus, novel diagnostic or prognostic biomarkers and therapeutic targets for HCC are urgently required. In the present study, we discovered that elevated levels of CEP55 are highly associated with vascular invasion, histologic grade, TNM stage, and serum AFP levels, strongly supporting the hypothesis that CEP55 might play significant roles in the progression of human HCC. Moreover, elevated expression of CEP55 mRNA is an independent prognostic factor in the reduction of OS and DFS rates in HCC patients. Taken together, our findings indicate that elevated expression of CEP55 is correlated with tumor invasiveness and may be an independent prognostic factor for clinical outcomes in human HCC patients.

A variety of aberrantly-expressed genes in cancer cells are involved in the regulation of cell migration and invasion. These genes need to be further validated as potential therapeutic targets. However, in human HCC, the molecules and mechanisms involved in governing cell migration and invasion remain largely unknown. CEP55 was initially characterized as a centrosome- and mid-body-associated protein, which plays significant roles in regulating physical cytokinesis. Interestingly, several studies have illuminated that CEP55 is involved in the process of cell mobility and metastasis of cancers [6]. For instance, upregulated expression of CEP55 was reported to modulate the invasion and migration of lung cancer [10], nasopharyngeal carcinomas [19], and oral cavity squamous cell carcinoma [8]. These elegant findings strongly imply the significance of CEP55 in cancer propagation. However, the biological function of CEP55 in supporting cancer cell spreading in HCC cancer has not been determined. Our results recommended that CEP55 may serve as a predictor for human HCC prognosis. Additionally, the present study indicated that CEP55 supports HCC cell invasion and migration through regulating JAK2–STAT3–MMPs signaling. Taken together, our results may give rise to a development in the field of CEP55 biological function in promoting HCC cancer cell motility. Cell migration and invasion are directly associated with cancer metastasis; therefore, further studies are needed to confirm if and how CEP55 supports HCC metastasis in vivo by using orthotropic xenograft models. In addition, the effect of CEP55 on human cancers needs to be fully verified in clinical research.

Over the past decade, intensive research has been undertaken to understand the function and molecular mechanism of CEP55 in the context of cancers. It was reported that CEP55 binds to PIK3CA and promotes PI3K/AKT pathway activation [7]. Recently, Sinha et al. showed that CEP55 directly binds to p110 subunit of PI3K in vivo and sustains activation of PI3K/AKT signaling [20]. Another study suggested that CEP55 regulates FOXM1 expression in a dose-dependent manner [8]. Furthermore, CEP55 enhanced glucose metabolism by controlling the expression levels of GLUT1 in glioma cells [21]. Notably, CEP55 was shown to support the invasion and metastasis of cancer cells via upregulating MMP2 expression [8,22]. In this study, we first identified that the JAK2–STAT3 pathway, but not JAK1–STAT3, is a novel responsive component of CEP55. Importantly, we firstly found that CEP55 physiologically interacts with JAK2 in vitro and promotes IL-6-induced JAK2 and STAT3 phosphorylation. These results demonstrate that targeting CEP55 might be a novel approach to selectively suppress the JAK2–STAT3–MMPs signaling pathway. Further studies are required to illuminate the molecular mechanisms by which CEP55 stimulates JAK2 phosphorylation and activation in vivo. Strikingly, the JAK/Stat signaling pathway has positioned itself as a promising therapeutic target to treat human cancers [23]. Thus, the first identification of JAK2–STAT3–MMPs axis as a new target of CEP55 might help to elucidate how CEP55 drives cancer propagation, in particular, its contribution to the design of effective therapeutic strategies for treating human HCC.

## 5. Conclusions

**Figure 7:**
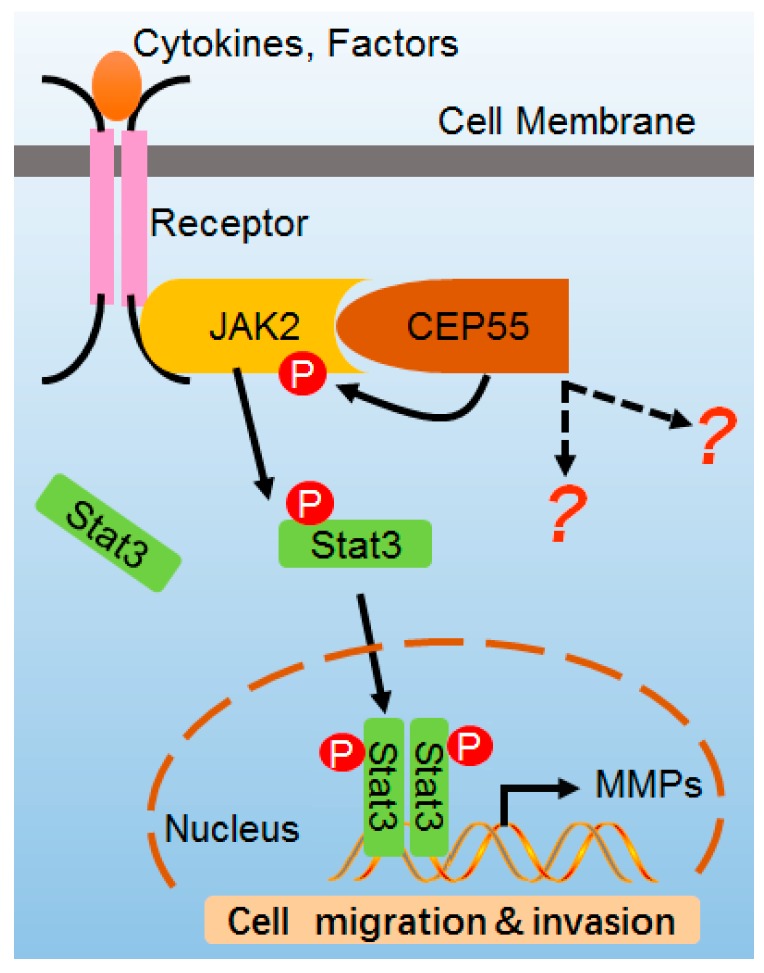
Model of CEP55 in JAK2–STAT3 signaling. CEP55 physiologically interacts with JAK2, leading to the heightening of JAK2 phosphorylation and activation induced by ligands, which activates JAK2 and then phosphorylates STAT3 which transfers into the nucleus, switching on the expression of MMP2/9 genes. Finally, CEP55 promotes the cell migration and invasion of HCC cells.

In summary, our findings suggest that CEP55 upregulation is an abnormal event in HCC and may support human HCC development. We showed that elevated CEP55 expression is correlated with HCC progression and poor prognosis of HCC patients. Additionally, overexpression of CEP55 in HCC cells contributes to the stimulation of the JAK2–STAT3–MMPs axis and, hence, to the induction of HCC cell migration and invasion (Figure 7). Future studies of physiological targets of CEP55 and its potential function in the pathogenesis of HCC will facilitate the development of effective therapeutic strategies for human HCC treatment.

## Figures and Tables

**Table 1:** Correlation of CEP55 transcription with clinicopathologic features of HCC patients.

Variable	CEP55 mRNA Expression ^#^		*p*-Value (χ^2^)
	Low	High	
Gender			
Male	86	75	0.1540
Female	30	41	
Age (year)			
≤50	18	30	0.0739
>50	98	86	
AFP (ng/mL)			
≤200	101	69	<0.0001 *
>200	15	47	
Hepatitis status			
Negative	55	47	0.3545
Positive	61	69	
Vascular invasion			
No	91	72	0.0095 *
Yes	25	44	
Histologic grade			
I	19	5	<0.0001 *
II	64	43	
III–IV	33	68	
TNM stage			
I	74	52	0.02 *
II	22	30	
III–IV	20	32	

**^#^** A median split was performed. Fisher’s exact test. * *p*-Values showing a significant difference. The data were obtained from Cbioportal.

## Supplementary Materials

Supplementary Materials are available online at http://www.mdpi.com/2073-4409/7/8/99/s1.
