# Supplementary material for: CEP55 Promotes Cell Motility via JAK2–STAT3–MMPs Cascade in Hepatocellular Carcinoma
Source: Cells. 2018 Aug 8;7(8):99. doi: 10.3390/cells7080099 (PMC6115913; doi:10.3390/cells7080099)
Supplement: Supplementary file 1 [file cells-07-00099-s001.zip › cells-320182-SI.pdf]

Supplementary Materials

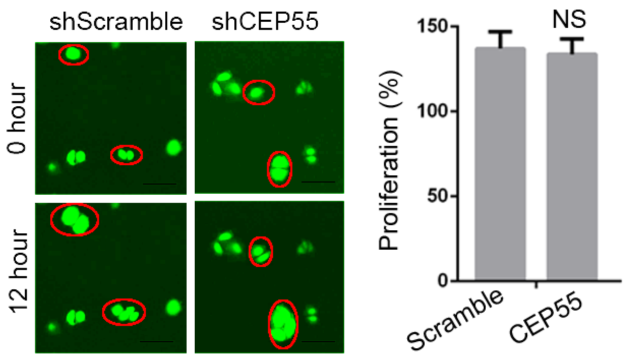

Figure S1. Knockdown expression of CEP55 has no effects on cell proliferation within 12 h. HepG2 cells at were infected with Scramble or CEP55 shRNA for 48 h. Then 10,000 cells were seeded into 12 well plates for proliferation assays. Shown are representative images at indicated hours after cell adherence (**left panel**) and data summary (**right panel**). N = 3, NS, no significant difference. Bars, 50 μm.

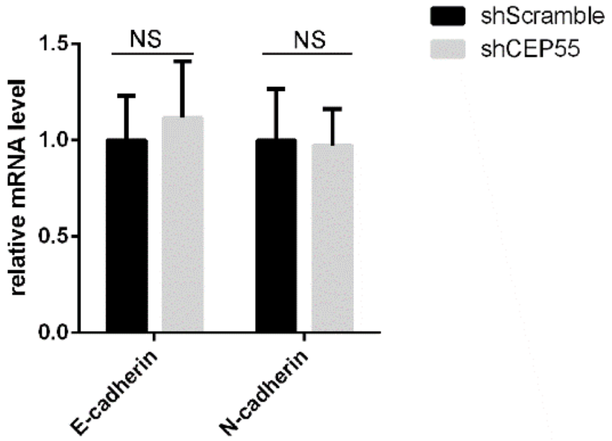

Figure S2. Knockdown expression of CEP55 has no effect on EMT marker transcription. HCC cells were transfected with indicated shRNA for 24 h followed by real-time-PCR analysis of E-cadherin and N-cadherin transcription. N = 3, NS, no significant difference.

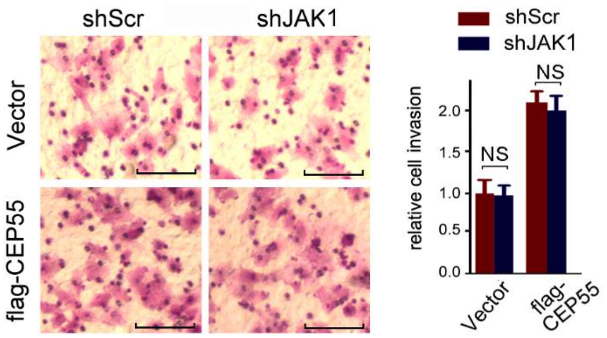

Figure S3. JAK1 does not affect CEP55 induced invasion of HCC cells. flag-CEP55 were transfected into Hep3B cells, 24 h later cells were treated with Scramble or JAK1 shRNA for 48 h. Then invasion ability was analyzed by trans-well assay. Representative images were plotted Bars, 50 μm (**left panel**). Relative invasion cell number of Hep3B cells was plotted (**right panel**). Cell number in control group was set as 1. N = 4, NS, no significant difference.

Cells **2018**, *7*, 99; doi:10.3390/cells7080099 www.mdpi.com/journal/cells
